# A Behavior Change Technique–Based Text Messaging Intervention for Obesity Among Employed Adult Men Younger Than 40 Years: Pilot Randomized Controlled Trial

**DOI:** 10.2196/104711

**Published:** 2026-10-20

**Authors:** Chie Tomotake, Yoshio Ohyama

**Affiliations:** 1 Department of Nursing, Faculty of Nursing, Mejiro University, 320 Ukiya, Iwatsuki-ku, Saitama-shi, Saitama, 339-8501, Japan, 81 48-797-2111; 2 Department of Nursing, Graduate School of Health Sciences, Gunma University, Maebashi, Gunma, Japan

**Keywords:** obesity, SMS text messaging, behavior change techniques, mobile health, randomized controlled trial, workplace health promotion

## Abstract

**Background:**

In Japan, employed men younger than 40 years are not eligible for specific health guidance, and evidence regarding obesity interventions targeting this population is limited. Furthermore, due to the shortage of human resources in occupational health settings, a need has arisen for simple and implementable support methods. Support via SMS text message is expected to be a low-cost and scalable method for health guidance; however, its effectiveness in this target population has not been sufficiently evaluated.

**Objective:**

This pilot randomized controlled trial aimed to assessed the feasibility, acceptability, and preliminary effectiveness of an SMS text messaging intervention among employed men younger than 40 years with obesity (BMI ≥25 kg/m²).

**Methods:**

An unblinded randomized controlled trial was conducted with 35 participants (intervention group, n=17; control group, n=18). The intervention group received one-way SMS text messaging based on behavior change techniques targeting diet and physical activity, delivered 3 times per week for 12 weeks (36 messages total). The control group received a pamphlet and a self-monitoring record sheet at baseline. The primary outcome was the proportion achieving ≥3% weight loss. Secondary outcomes included BMI, waist circumference, and psychological and behavioral measures. Feasibility and acceptability were also assessed.

**Results:**

The proportion achieving ≥3% weight loss was 23.5% (4/17) in the intervention group and 5.6% (1/18) in the control group (*P*=.18; odds ratio 5.23). Waist circumference significantly decreased in the intervention group compared to baseline (*t*_16_=2.71, *P=.*02), whereas no significant between-group differences were observed in weight, BMI, or psychological measures. The SMS delivery rate was 95.5%, with a total cost of JP¥ 15,264 over 12 weeks. The follow-up rate was 100%, and the ratings for satisfaction, utilization, and willingness to recommend were 3.5, 2.8, and 3.0 out of 5, respectively.

**Conclusions:**

The findings support the feasibility and acceptability of an SMS text messaging intervention for health guidance among employed men younger than 40 years with obesity, although evidence of its effectiveness remains preliminary. Further research with larger samples is needed to evaluate its effectiveness and optimize the intervention.

## Introduction

Obesity is a major global health issue, with projections suggesting that by 2035, approximately 50% of the world’s population will be overweight or obese [1]. In Japan, given the risks of obesity-related health problems, a BMI of 25 kg/m² or higher is classified as obesity [2]. This differs from the World Health Organization classification, which defines obesity in adults as a BMI of 30 kg/m² or higher [3]. Obesity in men is characterized by its onset in young adulthood. According to the 2024 National Health and Nutrition Survey conducted by the Japanese Ministry of Health, Labor and Welfare, the prevalence of obesity (BMI ≥25 kg/m²) among Japanese adults was 25.3%; among men aged 20‐29 and 30‐39 years, the rates were 21.0% and 34.3%, respectively [4]. These findings suggest that intervention from young adulthood through early middle age is crucial. Factors contributing to obesity in this age group include work-related stress, irregular eating habits, and physical inactivity [5].

In individuals with obesity, the accumulation of visceral fat increases the risk of metabolic disorders and atherosclerotic diseases; however, weight loss achieved through lifestyle modifications can improve blood glucose levels, lipid levels, and blood pressure [2]. Therefore, interventions supporting lifestyle changes are crucial for the prevention and management of obesity.

In Japan, specific health checkups and specific health guidance are conducted for individuals aged 40 years and older, and certain improvements have been reported [6,7]. Some reports also indicate that improvements in cardiovascular risk factors other than obesity have been limited [8]. On the other hand, health management support for employed men younger than 40 years, who are not eligible for specific health checkups, is primarily left to voluntary corporate initiatives; however, due to a shortage of occupational health professionals, many workplaces have difficulty providing sufficient support [9]. Evidence for obesity interventions targeting employed men younger than 40 years is also scarce. To the best of our knowledge, obesity intervention studies targeting young employed men are limited to a single report combining website use with face-to-face support [10]. Against this backdrop, there is a need to develop simple and feasible intervention methods for this population.

In recent years, the spread of information and communication technology has led to the expansion of lifestyle improvement support using mobile health (mHealth). In obesity interventions, support using text messages has been reported to offer implementation advantages over app-based interventions, such as lower dropout rates and higher reach and open rates [11,12]. Although the effectiveness of SMS text messaging–based obesity interventions in adults has been demonstrated [13], the same review identified a lack of sufficient analysis of the sex-specific effects of SMS text messaging interventions as a key challenge. Men are significantly underrepresented in obesity intervention studies [14,15]; to the best of our knowledge, no randomized controlled trials (RCTs) have evaluated SMS text messaging as the primary intervention method for employed men younger than 40 years.

Behavior change techniques (BCTs) constitute an internationally standardized framework based on intervention components common to numerous existing health behavior theories [16,17]. BCTs have been incorporated into text message interventions for various health issues and applied to improve physical activity and dietary habits [18-21]. The Game of Stones Trials is an example of a text message intervention designed to explicitly incorporate BCTs and target individuals with obesity. In this trial, narrative-style text messages incorporating BCTs were developed and evaluated in a multiarm parallel-group randomized controlled trial involving men with obesity aged 18 years or older with a BMI of 30 kg/m² or higher [22]. However, this study targeted the general adult male population, and findings on employed men younger than 40 years are limited.

Therefore, this pilot randomized controlled trial aimed to assess the feasibility, acceptability, and preliminary effectiveness of an SMS text messaging intervention among employed men younger than 40 years with obesity.

## Methods

### Overview

This pilot randomized controlled trial was conducted to evaluate the feasibility, acceptability, and preliminary effectiveness of an SMS text messaging intervention focused on lifestyle self-management for weight management among employed men younger than 40 years.

To strengthen the theoretical basis of the intervention, this study incorporated internationally standardized BCTs into an SMS intervention in Japan.

Participants were randomly assigned to the intervention or control group. Owing to the nature of the intervention, neither the participants nor the principal investigator could be blinded to group allocation.

### Population

The inclusion criteria were as follows: male workers aged 18‐39 years with a BMI of ≥25 kg/m², possession of a smartphone or mobile phone and ability to use SMS text messaging services and access the internet, ability to read and write Japanese and understand the study content, and participation in workplace health checkups. The exclusion criteria were as follows: work involving night shifts and current medical treatment for a pre-existing condition that prevents participation in healthy eating or physical activity for weight management. Workers engaged in night-shift work were excluded to reduce heterogeneity related to work schedules and associated differences in lifestyle patterns, such as eating and sleeping patterns, in this pilot study.

### Recruitment and Procedure

Occupational physicians and occupational health nurses recruited participants from their workplaces using written or email invitations and workplace postings, as appropriate to each workplace. The participating workplaces were identified through the professional networks of the principal investigator and a coinvestigator. The researchers did not directly recruit employees. The recruitment period extended from December 2024 to April 2025 because the planned number of participants was not reached within the initial recruitment period. Participants who met the inclusion criteria received a QR code on the study website and provided informed consent.

After obtaining consent, the occupational health nurses measured the participants’ weights and waist circumferences at the participating workplaces. Body weight was measured using a TANITA BC-315 body composition scale (Tanita Corporation). Waist circumference was measured using a seca 201 ergonomic circumference measuring tape (seca GmbH & Co KG). Height data for the BMI calculations were obtained from workplace health examination records. The participants completed a questionnaire, including demographic characteristics, using Google Forms. Follow-up assessments were completed in August 2025.

### Randomization

Participants were randomly assigned to the intervention or control group in a 1:1 ratio using permuted block randomization with a block size of 2. Participants from the same workplace could be assigned to either the intervention or control group. Randomization was implemented using a Microsoft Excel–based tool incorporating the RAND function, which was prepared and provided by a statistician who was not involved in participant recruitment. After informed consent had been obtained and participant enrollment was completed, the principal investigator entered each participant into the randomization tool, which generated the group assignment. Thus, group allocation remained unknown until after participant enrollment. Owing to the nature of the intervention, neither the participants nor the principal investigator could be blinded to group allocation. However, the occupational health nurses who performed the outcome measurements were not informed of participants’ group allocation. The principal investigator conducted the statistical analyses and was aware of group allocation.

### Interventions

#### Intervention Group

At the start of the study, participants were given the March 2023 versions of the “Lifestyle Habits Review Notebook for Healthy Body Building (Men’s Edition)” and the “Lifestyle Improvement Record Sheet,” developed by the Japanese Ministry of Health, Labor and Welfare [23], to help them review and monitor their lifestyle habits. The pamphlet contained information on obesity and metabolic syndrome, physical activity and dietary habits, strategies for increasing physical activity and modifying dietary habits, weight-loss goal setting, and self-monitoring. The record sheet included fields for recording body weight, step count, two lifestyle improvement goals and their achievement, weekly satisfaction with their efforts, and comments. Over a 12-week period, health guidance on the self-management of lifestyle habits for weight management was provided via SMS text messaging 3 times per week.

The technical platform used for sending text messages was the SMS messaging service provided by CM.com Japan Co Ltd. The intervention consisted of one-way SMS text messaging, and the participants were not asked to reply.

Text messages were created by the principal investigator and the coinvestigator. The content of the messages addressed lifestyle behaviors and related factors relevant to weight management, including diet, alcohol consumption, physical activity, sleep, psychosocial and socioeconomic factors, and self-monitoring. These lifestyle domains were based on the 2022 Clinical Practice Guidelines for Obesity [2] and the Standard Health Checkup and Health Guidance Program (FY 2024 Edition) [24] (Table S1 in Multimedia Appendix 1). The intervention was not designed to prescribe a specific reduction in energy intake or increase in energy expenditure to achieve a predetermined energy deficit. To support behavior change within these lifestyle domains, 10 BCTs commonly identified across all 6 review articles included in our previously published narrative review [25] were used as the basis for the intervention design (Table S2 in Multimedia Appendix 1). During message development, the principal investigator primarily selected the BCT(s) to be incorporated into each message, and their appropriateness was reviewed with a coinvestigator. Neither researcher had completed formal BCT coding training. Independent BCT coding and formal assessment of interrater agreement were not conducted. Based on these lifestyle domains and BCTs, short messages and quiz-style messages were developed. The message content remained unchanged during the intervention. Multimedia Appendix 2 presents all text messages delivered during the intervention, together with the corresponding lifestyle domain(s) and BCT(s) for each message. URLs of web pages were included to provide additional explanations as needed. Messages were delivered 3 times per week (Mondays, Wednesdays, and Fridays) between 12:00 PM and 1:00 PM.

During the development of the text messages, we solicited feedback on the draft messages from 5 research collaborators who were occupational health nurses and 4 men in their 20s and revised the text based on their input. The occupational health nurses reviewed the messages for accuracy, readability, and tone. As part of the formative evaluation, all text messages were sent to 4 men in their 20s to assess the clarity and readability of the messages, identify wording that they considered inappropriate or unclear, evaluate the appropriateness of the sending times, and ensure that there were no issues with transmission or reception. Additionally, to ensure the stable implementation of the intervention, a system was established in advance, whereby researchers could send messages directly from their research smartphones in the event of delivery problems.

#### Control Group

At the start of the study, participants were provided with the same pamphlet and record sheet as those provided to the intervention group. No additional text messages were sent during the intervention.

### Outcome

#### Primary Outcome

The effectiveness of the intervention was assessed based on the weight loss achievement rate from baseline to the 3-month follow-up assessment.

Weight loss achievement was defined as a reduction of 3% or more of baseline body weight. The 2022 Clinical Practice Guidelines for Obesity recommend a reduction of at least 3% of current body weight as the initial weight-loss target and evaluation of treatment outcomes over 3‐6 months [2]. A binary outcome was selected as the primary outcome to evaluate whether individual participants achieved this clinically relevant weight-loss threshold. As a supplementary analysis, the change in body weight (postintervention minus baseline) was calculated, and differences between the groups were evaluated using a 2-tailed independent samples *t* test (assuming equal variance).

#### Secondary Outcomes

BMI, waist circumference, the Weight Efficacy Lifestyle Questionnaire (WEL), the Japanese version of the New General Self-Efficacy Scale (NGSE-J), and the stage of behavior change regarding efforts to improve lifestyle habits were assessed at baseline and at the 3-month follow-up.

WEL [26] is a scale designed to measure self-efficacy regarding energy intake restrictions in individuals with obesity. The WEL consists of 20 items rated on a 10-point scale ranging from 0 (not at all confident) to 9 (very confident). In this study, item scores were summed for each participant to calculate a total score ranging from 0 to 180, with higher scores indicating greater self-efficacy for resisting eating. The WEL used in this study was based on the original English version. Although the reliability and validity of the Japanese version had been verified at the time of this study [27], the details of the items have not yet been published. Therefore, after confirming the situation with the authors of the Japanese version, the research team created a Japanese translation based on the original version, which was used after the content had been verified by a coinvestigator of the present study. The measurement results from this scale were used as exploratory indicators.

The NGSE-J measures general self-efficacy and is not limited to specific domains. We used the Japanese version [28,29], for which the reliability and validity had been verified and published at the time of this study. The NGSE-J consists of 8 items rated on a 5-point scale ranging from 1 (strongly disagree) to 5 (strongly agree). In this study, item scores were summed for each participant to calculate a total score ranging from 8 to 40, with higher scores indicating greater general self-efficacy.

The stage of behavior change regarding efforts to improve lifestyle habits was assessed based on the Ministry of Health, Labor and Welfare’s Standard Health Checkup and Health Guidance Program (FY 2024 Edition) [24]. In this program, responses regarding lifestyle improvements were categorized into five stages: (1) no intention to improve, (2) intention to improve (generally within 6 mo), (3) intention to improve within 1 month, (4) already implementing (less than 6 mo), and (5) continuous implementation for 6 months or more. These stages correspond to indifference, interest, preparation, action, and maintenance, respectively. Therefore, this study treated these stages as a 5-point ordinal scale. The assessment consisted of 7 items. Scores for the 7 items were summed for each participant to calculate a total score ranging from 7 to 35 at baseline and at the 3-month follow-up. The change score was calculated by subtracting the baseline total score from the postintervention total score.

#### Feasibility Measures

As feasibility measures, we assessed the SMS delivery rates and URL click-through rates at baseline, 1, 2, and 3 months.

Additionally, as one of the measures used to evaluate the feasibility of the intervention, we calculated the cost incurred to deliver the 12-week intervention. The cost calculation included only SMS delivery fees, calculated from the total number of SMS units sent and the per-unit fee. Personnel costs and other research-related expenses were not included.

#### Acceptability Measures

Acceptability of the SMS text messaging intervention was evaluated exclusively among participants in the intervention group. At 3 months, satisfaction with the SMS message content, utilization of the SMS messages, and willingness to recommend the intervention to peers of a similar age with obesity were assessed via an online survey using 5-point scales. Satisfaction was rated from 1 (“very dissatisfied”) to 5 (“very satisfied”), utilization from 1 (“not used at all”) to 5 (“used to a great extent”), and willingness to recommend the intervention from 1 (“would not recommend at all”) to 5 (“would highly recommend”). The appropriateness of SMS delivery frequency, timing, and duration was assessed using 2 response options (“not at all appropriate” and “very appropriate”).

### Sample Size

No formal a priori power calculation for intervention effectiveness was performed because the study was planned as a pilot randomized controlled trial. As recorded in the University Hospital Medical Information Network Clinical Trials Registry (UMIN-CTR; trial ID UMIN000054203), the initial target sample size was 90 participants, with 45 participants allocated to each group. The target was calculated to obtain approximately 30 evaluable participants per group after allowing for an anticipated attrition rate of 30% (30/0.70=42.9), which was rounded up to 45 participants per group. The anticipated attrition rate was based on previous mHealth weight-management studies that anticipated attrition rates of 20% and approximately 30%, respectively [30,31].

Owing to recruitment constraints during the planned study period, 35 participants were ultimately randomized, including 17 in the intervention group and 18 in the control group. A sensitivity analysis based on the achieved sample size indicated that, with a 2-sided α of .05 and 80% power, the minimum detectable standardized effect size for the binary primary outcome was approximately Cohen h=0.95. Although no participants were lost to follow-up, the randomized sample remained below the planned target; therefore, findings regarding intervention effectiveness should be interpreted as exploratory.

### Statistical Analysis

Statistical analyses were performed using IBM SPSS Statistics (version 31.0.1.0). Analyses were conducted according to the intention-to-treat (ITT) principle and included all randomized participants. When missing values were present, a per-protocol set (PPS) analysis was also used.

For the intervention and control groups, baseline age (years), height (cm), BMI (kg/m²), and waist circumference (cm) were calculated as mean (SD), and intergroup comparisons were performed using a 2-tailed independent samples *t* test.

Differences in the primary outcome, defined as the proportion of participants achieving a weight loss of 3% or more, were compared between the intervention and control groups using a *χ*² test. The Fisher exact test was applied when the expected frequency of any cell was ≤5. Odds ratios and 95% CIs were calculated to estimate effect sizes. Because binary classification of the achievement rate may result in a loss of information, weight change (postintervention minus baseline) was calculated as a supplementary analysis, and group differences were evaluated using a 2-tailed independent samples *t* test (assuming equal variances).

For secondary outcomes, 2-tailed paired *t* tests were performed to evaluate the changes in BMI and waist circumference from baseline to postintervention within each group. For the WEL, NGSE-J, and behavior change stages regarding lifestyle improvement efforts, the Mann-Whitney *U* test was used to compare the intervention and control groups regarding the magnitude of change from baseline to postintervention, as well as the postintervention measurements. As the WEL, NGSE-J, and behavior change stages regarding lifestyle improvement efforts did not follow a normal distribution, descriptive statistics were presented as medians and interquartile ranges.

Evaluations of satisfaction, utilization, and willingness to recommend SMS text messages (sent only to the intervention group), as well as the frequency, timing, and duration of delivery, were presented using descriptive statistics. SMS delivery was summarized using frequency counts and delivery rates, and URL click-through rates were summarized as percentages. Descriptive statistics were used to calculate the costs associated with SMS text messaging during the delivery period.

Because this pilot study was designed to assess feasibility, acceptability, and preliminary effectiveness rather than to provide definitive evidence of effectiveness, we calculated the effect size (odds ratio) and 95% CI for the primary outcome, in addition to hypothesis testing, to evaluate the magnitude and precision of the intervention effect. For all statistical analyses, the significance level was set to 5% (2-tailed).

### Ethical Considerations

This study was approved by the Medical Research Ethics Review Committee of Mejiro University in March 2023 (approval number 23-I-054). The trial was prospectively registered in the UMIN-CTR (trial ID UMIN000054203). We adhered to the CONSORT-EHEALTH (Consolidated Standards of Reporting Trials of Electronic and Mobile Health Applications and online Telehealth) guidelines (see Checklist 1) [32] when reporting the study findings.

Participants were informed of the purpose and methods of the study, the voluntary nature of participation, and the protection of their personal information and were assured they would suffer no disadvantages if they declined to participate or withdrew from the study. Digital informed consent was obtained using the landing page service provided by CM.com, a provider of SMS messaging services. All collected data were anonymized before analysis to ensure participants’ confidentiality and privacy. All participants who completed the study were provided with a gift card worth JP¥ 2000 (approximately US $12.50) as a token of appreciation.

## Results

### Participant Flow and Baseline Characteristics

A total of 43 participants were screened in the study. Seven participants who did not meet the inclusion criteria and one who withdrew from the study were excluded. In all, 35 participants were included in the study and randomly assigned to the intervention group (n=17) or the control group (n=18). During the follow-up period, all 17 participants in the intervention group completed the 12-week intervention, and all 18 participants in the control group completed the trial. No participants dropped out in either group. The demographic and work-related characteristics of the participants are presented in Table 1.

**Table 1.** Demographic and work-related characteristics of participants at baseline.

Variables	Total (N=35), n (%)^a^	Intervention group (N=17), n (%)^a^	Control group (N=18), n (%)^a^
Age (years)			
Teens	1 (3)	0 (0)	1 (6)
20s	13 (37)	8 (47)	5 (28)
30s	21 (60)	9 (53)	12 (67)
Marital status			
Married	18 (51)	8 (47)	10 (56)
Unmarried/divorced/widowed	17 (49)	9 (53)	8 (44)
Job type			
Professional and technical	15 (43)	7 (41)	8 (44)
Clerical	5 (14)	2 (12)	3 (17)
Sales	6 (17)	3 (18)	3 (17)
Service industry	1 (3)	0 (0)	1 (6)
Production	4 (11)	2 (12)	2 (11)
Transportation and cleaning	1 (3)	1 (6)	0 (0)
Planning	2 (6)	1 (6)	1 (6)
Teacher	1 (3)	1 (6)	0 (0)
Working hours per week			
29 hours or less	3 (9)	1 (6)	2 (11)
35‐39 hours	12 (34)	2 (12)	10 (56)
40‐48 hours	17 (49)	12 (71)	5 (28)
49‐59 hours	3 (9)	2 (12)	1 (6)

a Percentages may not total 100 because of rounding.

Baseline data were analyzed for all randomized participants. For psychological and behavioral measures, because 4 participants in the control group had missing responses, the baseline values were imputed for missing data in the ITT analysis. In the PPS analysis, only participants with complete responses were included (Figure 1). Physical, psychological, and behavioral indicators at baseline are presented in Table 2.

**Figure 1.**
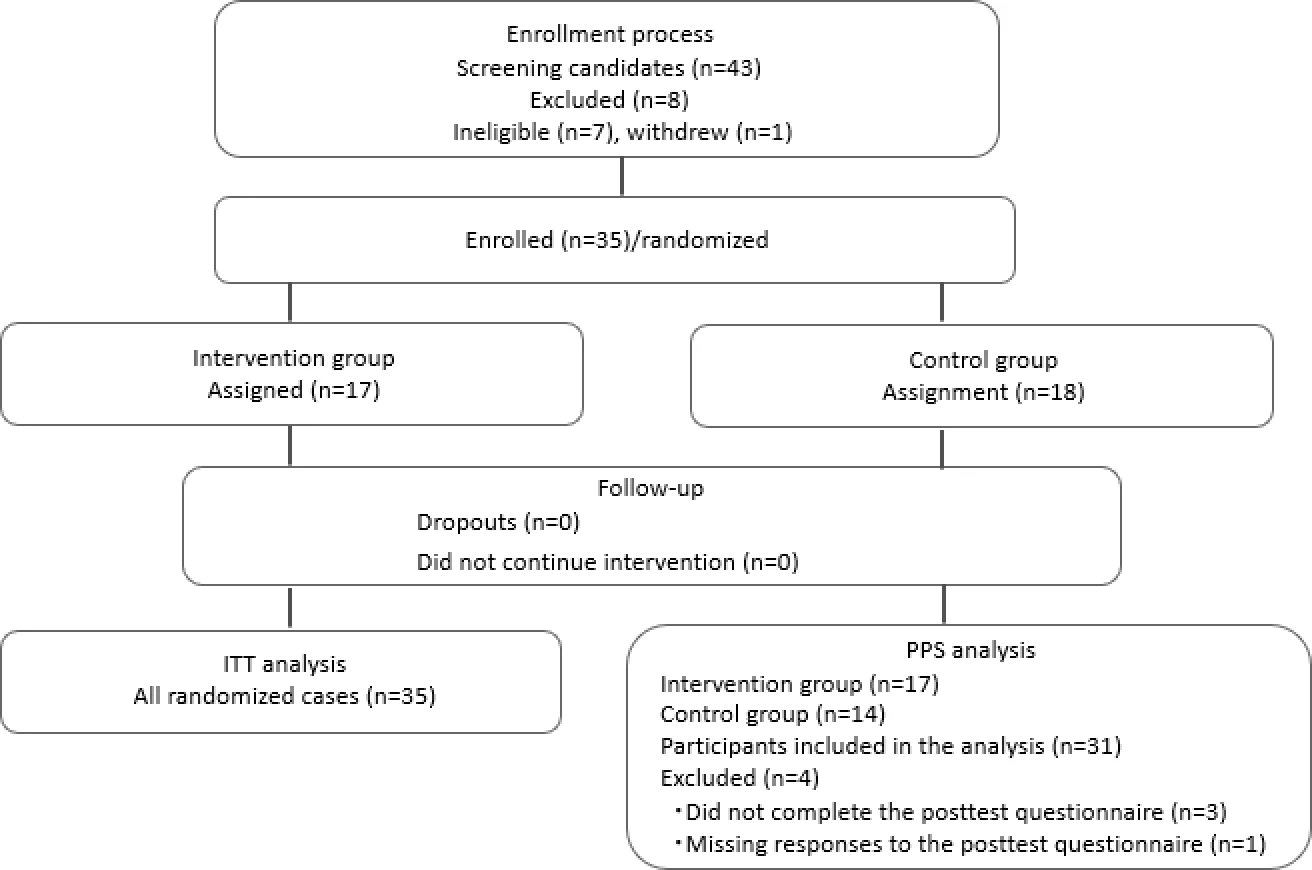
Participant flow from enrollment through analysis in the pilot randomized controlled trial. ITT: intention-to-treat; PPS: per-protocol set.

**Table 2.** Physical, psychological, and behavioral indicators at baseline.

	Intervention group (N=17)	Control group (N=18)
Age (years), mean (SD)	30.9 (4.4)	30.0 (4.7)
Height (cm), mean (SD)	172.5 (4.9)	171.1 (5.5)
Weight (kg), mean (SD)	88.1 (14.6)	84.6 (8.3)
BMI (kg/m²), mean (SD)	29.5 (4.4)	28.8 (1.8)
Waist circumference (cm), mean (SD)	99.6 (10.0)	97.3 (6.2)
WEL^a^ total, median (IQR)	87.0 (79.0‐113.0)	83.0 (65.0‐104.0)
NGSE-J^b^ total, median (IQR)	21.0 (16.0‐25.0)	23.5 (16.0‐30.0)
Behavior change stage, median (IQR)	17.0 (15.0‐18.0)	17.0 (14.0‐19.0)

a WEL: Weight Efficacy Lifestyle Questionnaire.

b NGSE-J: Japanese version of the New General Self-Efficacy Scale.

### Primary Outcome

A greater proportion of participants in the intervention group than in the control group achieved at least 3% weight loss, but the between-group difference was not statistically significant. The supplementary analysis of weight change likewise showed no significant between-group difference (Table 3).

### Secondary Outcomes

#### Physical, Psychological, and Behavioral Measures

BMI showed no significant within-group change in either group. Waist circumference decreased significantly from baseline in the intervention group, whereas no significant change from baseline was observed in the control group (Table 4).

**Table 3.** Weight loss outcomes: 3% weight loss achievement rate and weight change outcomes.

	Intervention group (N=17)	Control group (N=18)	Between-group difference or effect size	*t* test (*df*)	*P* value
Primary outcome					
Participants achieving 3% weight loss, n/N (%)	4/17 (23.5)	1/18 (5.6)	Odds ratio 5.23 (0.52 to 52.55)^a^	N/A^b^	.18
Supplementary analysis					
Change in body weight, kg, mean (SD)	−1.61 (3.53)	−0.23 (2.25)	−1.37 (−3.40 to 0.65)^c^	1.38 (33)	.18

a Estimated effect (95% CI).

b Fisher exact test was used for the between-group comparison.

c Between-group difference (95% CI).

**Table 4.** Changes in BMI and waist circumference from baseline to 3 months by study group.

Item and group		Preintervention, mean (SD)	3 months later, mean (SD)	*t* test (*df*)	*P* value
BMI					
	Intervention group (N=17)	29.5 (4.4)	29.0 (4.5)	1.81 (16)	.09
	Control group (N=18)	28.8 (1.8)	28.7 (1.9)	0.58 (17)	.57
Waist circumference					
	Intervention group (N=17)	99.6 (10.0)	96.6 (11.5)	2.71 (16)	.02
	Control group (N=18)	97.3 (6.2)	95.9 (7.2)	1.46 (17)	.16

No significant differences were observed between the intervention and control groups regarding the postintervention total scores and change scores for the WEL and NGSE-J in the ITT and PPS analysis. Similarly, no significant differences were observed in the postintervention stages of behavior change or change scores in either the ITT or PPS analysis (Table S4 in Multimedia Appendix 3).

#### Feasibility Measures

During the intervention period, excluding test messages and administrative notifications, SMS delivery remained high. Nondelivery tended to recur among specific participants; however, the exact reasons for nondelivery could not be determined from the available delivery records (Table 5).

**Table 5.** SMS delivery, URL click-through rates, and delivery cost during the 12-week intervention.

Item	Baseline	0‐1 month (cumulative)	0‐2 months (cumulative)	0‐3 months (cumulative)
Total number of SMS units	54	645	957	1350
Total SMS delivered	49	617	924	1289
Total undelivered SMS	5	28	33	61
Delivery rate (%)	90.7	95.7	96.6	95.5
URL click-through rate (%)	―^a^	36.6	34.1	28.7
Total cost (including tax)	―	―	―	JP¥^b^ 15,264 (US $97.41)

a Not applicable.

b JP¥: Japanese yen. Currency conversions were based on the exchange rate as of May 10, 2026.

The overall click-through rate for URLs embedded in the text messages was relatively limited (Table 5).

The reported SMS count represents the number of SMS units recorded by the delivery system rather than the number of intervention messages delivered. Messages exceeding 70 characters were counted as multiple SMS units; therefore, the baseline count shown in Table 5 represents SMS units rather than separate intervention contacts.

The total cost of SMS delivery during the 12-week intervention is reported in Table 5.

### Acceptability

At the 3-month follow-up, process evaluation data were obtained from all 17 participants in the intervention group, with no missing data. The mean (SD) ratings for satisfaction, utilization, and willingness to recommend were 3.5 (SD 1.1), 2.8 (SD 1.2), and 3.0 (SD 1.2), respectively (Table S5 in Multimedia Appendix 3). Delivery timing received less favorable ratings than those of delivery frequency and duration (Table S6 in Multimedia Appendix 3).

## Discussion

### Principal Findings

This pilot randomized controlled trial assessed the feasibility, acceptability, and preliminary effectiveness of an SMS text messaging intervention targeting lifestyle self-management among employed men younger than 40 years with obesity. The intervention was feasible in terms of SMS delivery and participant retention and was generally acceptable to participants. Regarding preliminary effectiveness, no statistically significant between-group differences were observed in the primary outcome or in the psychological and behavioral secondary outcomes. Waist circumference decreased significantly from baseline within the intervention group, whereas no significant within-group change was observed in BMI.

### Primary Outcome

No statistically significant between-group difference was observed in the primary outcome. Although the point estimate of the odds ratio favored the intervention group, the 95% CI was wide and included 1, indicating considerable uncertainty in the effect estimate.

Given the pilot design and limited sample size, the study was not designed to provide a definitive estimate of intervention effectiveness. Therefore, these findings regarding effectiveness should be interpreted as preliminary and require confirmation in an adequately powered trial.

In a similar worksite-based RCT involving employed men with obesity and a mean age of approximately 41 years, no significant between-group difference in weight loss was observed with the addition of SMS text messaging [33]. In that study, the intervention group received tailored SMS text messages in addition to face-to-face education, monthly weight measurements, and brief counseling, whereas in the present study, SMS text messaging was used as the primary mode of intervention. These findings suggest that further refinement of SMS content and delivery strategies may be needed to support weight loss among employed men with obesity.

### Secondary Outcomes

The reduction in waist circumference from baseline in the intervention group, despite the absence of a significant change in BMI, may reflect an early change that was not captured by body weight–based measures. Ross et al [34] reported that abdominal and visceral fat can decrease even in the absence of weight loss.

No between-group differences were observed in the psychological and behavioral outcomes, including WEL, NGSE-J, and stages of behavior change. Previous studies have suggested that SMS text messages can function as prompts and reminders to encourage health behaviors [12,35]; however, in the present study, these messages may have been insufficient to produce measurable changes in self-efficacy or stages of behavior change. The one-way nature of the intervention may also have limited opportunities for active participant engagement. Future studies should explore strategies to increase engagement, such as personalizing the text message content or introducing two-way communication.

### Potential Role of BCTs

The SMS text messages in this study incorporated 10 BCTs to support lifestyle behavior change. However, these BCTs were delivered through standardized, one-way SMS text messages, with limited opportunities to tailor the techniques to participants’ individual goals, behaviors, or progress. Similarly, in the Game of Stones trial, theory-based BCTs were incorporated into text messages for men with obesity, but text messaging alone did not result in significantly greater weight loss than the control condition [22]. Further research should examine how BCT delivery through SMS can be optimized to support behavior change.

### Acceptability and Feasibility of SMS Text Messaging Interventions

SMS text messaging showed a high delivery rate throughout the intervention period. A 12-month intervention study using SMS text messaging to support weight loss in individuals with obesity reported improvements in weight and waist circumference, even with relatively simple, regular SMS delivery alone [36]. Furthermore, while being a relatively simple method that does not involve interaction or face-to-face contact, it allows for the continuous provision of encouragement to improve lifestyle habits in the context of daily life [37]. As SMS text messaging does not require the installation of a dedicated app and can be received on mobile phones used in daily life, it is unlikely to impose a new burden on participants and has the potential to be easily used as a means of continuous support. These characteristics could enhance the feasibility of implementing workplace health guidance for employed men.

In contrast, the click-through rate for URLs included in text messages was relatively limited. Because the intervention involved one-way delivery via SMS text messaging and focused primarily on viewing health information, the participants may not have necessarily clicked on URLs. While the click-through rate is an indicator of the participants’ interest and engagement with the intervention content, it is not a direct indicator of the intervention’s effectiveness or behavior change. Given that the content of the text messages sent via SMS itself functions as support for behavior change, viewing the messages may have contributed to behavior changes. Follow-up was complete in the present study. The low-burden format, which does not require participants to reply or enter data, may have supported participant retention. This simplicity may have increased the acceptability of the intervention by busy working adults. Qualitative studies targeting young working adults have reported that time constraints associated with work act as barriers to practicing healthy eating and physical activity [38]. Therefore, SMS-based support, which is unlikely to impose a time burden, may be an intervention method that is easily accepted by working adults. Previous studies targeting men with obesity have reported the acceptability of interventions using story-based SMS [39,40]. Although messages in the present study were delivered between 12:00 PM and 1:00 PM so that participants could view them during their lunch break, this timing may not have suited all participants. Future interventions could therefore consider more flexible delivery times.

### Limitations

This study has some limitations. First, because this was a pilot study with a limited sample size, it was underpowered to detect significant differences between groups, limiting the certainty of the findings, particularly for the psychological and behavioral indicators. In addition, no adjustment for multiple testing was applied to the exploratory secondary outcomes; therefore, individual *P* values should be interpreted cautiously because of the type I error. Second, the Japanese version of the WEL used in this study has not yet been published, so these measurement results should be interpreted cautiously as exploratory findings. Third, blinding was not feasible, as participants in the intervention group were inherently aware they were receiving text messages. Furthermore, because participants from the same workplace could be assigned to either group and no specific measures were implemented to prevent the sharing of intervention messages, potential contamination between the intervention and control groups cannot be excluded. Complete follow-up should also be interpreted with caution, as the gift card provided after completion of the 3-month follow-up assessment may have contributed to participant retention.

Regarding the intervention itself, the reliance on one-way SMS text messages without individualized feedback or interactive support limited the overall intensity of the behavior-change support. Furthermore, while we measured SMS delivery and link click-throughs, the exact read rates of the messages and post-click on-page behavior could not be fully confirmed. Although the messages were grounded in BCTs, the study design did not allow us to isolate or verify the mechanisms of action of individual BCTs.

Finally, the short 12-week intervention period leaves the long-term maintenance of behavioral and weight changes unclear, and focusing exclusively on employed men younger than 40 years limits the generalizability of these findings. Future research should build on these exploratory results through adequately powered RCTs, longer follow-up periods, and study designs capable of clarifying the specific pathways and effects of individual BCTs.

### Conclusions

This pilot RCT supported the feasibility and acceptability of an SMS text messaging intervention among employed men younger than 40 years with obesity, while evidence of its effectiveness remains preliminary. This approach may therefore be useful for providing health guidance to this population. Larger studies are needed to evaluate effectiveness and optimize intervention content and delivery.

## Supplementary material

10.2196/104711 Multimedia Appendix 1 Lifestyle factors targeted by the intervention and behavior change techniques used.

10.2196/104711 Multimedia Appendix 2 List of SMS text messages sent to the intervention group (12 weeks, 3 times per week).

10.2196/104711 Multimedia Appendix 3 Exploratory psychological and behavioral outcomes and process evaluation measures.

10.2196/104711 Multimedia Appendix 4 Generative AI prompts and responses (in Japanese).

10.2196/104711 Checklist 1 CONSORT-EHEALTH checklist.

## Abbreviations

BCT: behavior change technique
CONSORT-EHEALTH: Consolidated Standards of Reporting Trials of Electronic and Mobile Health Applications and online Telehealth
ITT: intention-to-treat
mHealth: mobile health
NGSE-J: Japanese version of the New General Self-Efficacy Scale
PPS: per-protocol set
RCT: randomized controlled trial
UMIN-CTR: University Hospital Medical Information Network Clinical Trials Registry
WEL: Weight Efficacy Lifestyle Questionnaire
