# Supplementary material for: A Behavior Change Technique–Based Text Messaging Intervention for Obesity Among Employed Adult Men Younger Than 40 Years: Pilot Randomized Controlled Trial
Source: JMIR Form Res. 2026 Oct 20;10:e104711. doi: 10.2196/104711 (PMC13717270; doi:10.2196/104711)
Supplement: Supplementary file 1 [file formative-v10-e104711-s004.pdf]

Trial Registration: UMIN-CTR UMIN000054203；https://center6.umin.ac.jp/cgi-open-bin/ctr/ctr_view.cgi?recptno=R000061484　フローチャートを作成したい。ITT解析とPPS解析を行う。見本と枠組みのみでよい。

思考時間: 4 秒 ›

以下は、**CONSORT準拠のフローチャート（枠組み・見本）**です。**数値は入れず、箱（ボックス）構造のみ**にしています。
（介入：SMS等／ITT解析＋PPS解析の併記を想定）

見本1：ITT解析＋PPS解析を同一図内に併記（おすすめ）

```
【組入れ（Enrollment）】
┌──────────────────────────────┐
│ 適格性評価（Assessed for eligibility）  n=__ │
└──────────────────────────────┘
                 │
                 ▼
┌──────────────────────────────┐
│ 除外（Excluded）  n=__                      │
│  ・不適格（Not meeting criteria） n=__       │
│  ・参加辞退（Declined to participate） n=__  │
│  ・その他理由（Other reasons） n=__          │
└──────────────────────────────┘
                 │
                 ▼
【無作為割付（Randomized）】
┌──────────────────────────────┐
│ 無作為割付（Randomized） n=__               │
└──────────────────────────────┘
        ┌────────────────┴───────────────┐
        ▼                                ▼
【介入群】                              【対照群】
┌──────────────────────────┐   ┌──────────────────────────┐
│ 介入群に割付（Allocated） n=__ │   │ 対照群に割付（Allocated） n=__ │
└──────────────────────────┘   └──────────────────────────┘
        │                                │
        ▼                                ▼
┌──────────────────────────┐   ┌──────────────────────────┐
│ 介入の受領状況（Received） n=__  │   │ 介入/通常ケアの受領状況 n=__     │
│ 介入未受領（Did not receive） n=__│   │ 未受領（Did not receive） n=__    │
│  理由：______________________ │   │  理由：______________________ │
└──────────────────────────┘   └──────────────────────────┘
        │                                │
        ▼                                ▼
【追跡（Follow-up）】
┌──────────────────────────┐   ┌──────────────────────────┐
│ 追跡不能（Lost to follow-up） n=__│   │ 追跡不能（Lost to follow-up） n=__│
│  理由：______________________ │   │  理由：______________________ │
│ 介入中止（Discontinued） n=__    │   │ 中止（Discontinued） n=__        │
│  理由：______________________ │   │  理由：______________________ │
└──────────────────────────┘   └──────────────────────────┘
        │                                │
        ├───────────────┬────────────────┤
```

```
          │                │                       │
          ▼                ▼                       ▼
【解析（Analysis）】

◆ ITT解析（Intention-to-treat）
┌────────────────────────┐      ┌────────────────────────┐
│ ITT解析に含めた（Analyzed） n=__  │      │ ITT解析に含めた（Analyzed） n=__  │
│（割付後の全対象を原則含める）    │      │（割付後の全対象を原則含める）    │
└────────────────────────┘      └────────────────────────┘

◆ PPS解析（Per-protocol）
┌────────────────────────┐      ┌────────────────────────┐
│ PPS解析に含めた（Analyzed） n=__  │      │ PPS解析に含めた（Analyzed） n=__  │
│ PPS除外 n=__：理由（例）           │      │ PPS除外 n=__：理由（例）           │
│ ・介入未受領/逸脱/主要アウトカム欠測│      │ ・主要アウトカム欠測/重大逸脱 等   │
└────────────────────────┘      └────────────────────────┘
```

見本2：ITT用フローチャート（標準CONSORT型）＋PPSは別枠（簡潔）

2-1 ITT（標準）

- Enrollment → Excluded → Randomized → Allocated（2群）→ Follow-up → **Analyzed（ITT）**

2-2 PPS（別枠として図の下に小さく追記）

- 「PPS解析対象：各群n=（**PPS除外：各群n=**、理由：____）」
  ※"Protocol deviation / non-adherence / primary outcome missing" など、**PPS除外定義に沿って列挙**

枠に入れる「項目名」テンプレ（そのままコピペ可）

- 適格性評価（Assessed for eligibility）
- 除外（Excluded）：不適格／辞退／その他
- 無作為割付（Randomized）
- 割付（Allocated）：受領／未受領（理由）
- 追跡（Follow-up）：追跡不能（理由）／中止（理由）
- 解析（Analysis）**：ITT解析に含めた／PPS解析に含めた**／PPS除外（理由）

PPS除外理由（よく使う"箱の中の定型句"）

- 介入未受領（介入開始前に辞退 等）
- プロトコル逸脱（対象外治療の併用、重大な手順逸脱）
- 介入遵守不良（規定回数未満、規定期間未満）
- 主要アウトカム欠測（測定不能・未回答）
- 解析時点の条件不充足（例：評価時期が規定外）

必要なら、あなたの研究（SMS介入）向けに、**「PPSの定義（遵守基準）」を1行で箱に収まる表現**にして、フローチャートに埋め込める形まで整形します（数値は入れずに文言だけ）。

提出用ログ

本研究の実データは入力しません。JMIRの図表制限（本文5表以内を目安）に合わせて、本文中の「表3 減量アウトカム」表の見本（構造のみ）を提示してください。　　　　　　　この表には、主要アウトカムである3%減量達成率と、その解釈を補足する解析として体重変化量を含めたい。3%減量達成率は、介入群と対照群の群間比較にFisherの正確確率検定（両側）を用いた。体重変化量は「介入後値－ベースライン値」として算出し、群間差は「介入群－対照群」として、等分散を仮定した独立サンプルt検定により評価した。　日本語で述べてください。

思考時間: 2 〜 3 秒 ›

以下のように、**主要アウトカムと補足解析を1つの表に統合**すると、JMIRの本文表数を抑えつつ、結果の解釈もしやすくなります。

表3　減量アウトカム

アウトカム	介入群	対照群	群間差または効果量	95%信頼区間	P値
**主要アウトカム**					
3%減量達成者, n/N（%）			オッズ比		
**補足解析**					
体重変化量, kg, 平均（SD）			平均差		

表注案

注：3%減量達成は、ベースラインから介入後までに体重が3%以上減少した者と定義した。3%減量達成率の群間比較には、Fisherの正確確率検定（両側）を用いた。体重変化量は「介入後値－ベースライン値」として算出した。体重変化量の群間差は「介入群－対照群」として示し、等分散を仮定した独立サンプルt検定により評価した。

略語説明案

CI：confidence interval（信頼区間）
SD：standard deviation（標準偏差）

この表構造の意図

この表では、**3%減量達成率を主要アウトカムとして上段に置き、その解釈を補足する連続量アウトカムとして体重変化量を下段に配置**します。
統計手法は異なりますが、どちらも「減量アウトカム」に関する結果であり、主要アウトカムと補足解析として明確に区分すれば、1つの表にまとめて問題ありません。

表題は、以前の「主要アウトカムおよびその補足解析」でもよいですが、本文中の表名としては、**「表3 減量アウトカム」**の方が簡潔で使いやすいです。

提出用ログ

表4 RCTのアウトカムとし、介入群ならびに対照群の介入前後におけるBMIと腹囲の変化を示す表を作成したい。介入群（n=X）、対照群（n=Y）。対応のある t 検定を行った。枠組みのみ示す。数値の入力の誘導は不要。注の見本も不要

思考時間: 2 〜 3 秒 ›

以下のように、**群ごとに介入前・介入後・変化量・群内p値**を並べる形が最もわかりやすいです。対応のあるt検定なので、p値は各群内の「介入前 vs 介入後」の比較として示します。

表4　BMIおよび腹囲の介入前後の変化

アウトカム	群	n	介入前	介入後	変化量	p値
BMI, kg/m^2	介入群	X	平均値（SD）	平均値（SD）	平均値（SD）	p値
	対照群	Y	平均値（SD）	平均値（SD）	平均値（SD）	p値
腹囲, cm	介入群	X	平均値（SD）	平均値（SD）	平均値（SD）	p値
	対照群	Y	平均値（SD）	平均値（SD）	平均値（SD）	p値

この構造でよいと思います。
表4が「介入前後の群内変化」を示す表であれば、**効果推定値（95% CI）や群間差**は入れなくてもよいです。

JMIR Form Resへの投稿を計画している。Multimedia Appendix の構成を下記のようにしたい。構成の適切性について、意見を述べてください。JMIRの投稿規定は確認済みなので、アドバイスは不要。Consortと生成AIについては、別途計画しているので、アドバイスは不要。Multimedia Appendix 1；介入した生活習慣領域と使用数（元表1）、メッセージの基盤としたBCTsと使用数（元表2）、Multimedia Appendix 2：介入群に配信したショートメッセージ（SMS）の一覧（12週間、週3回）、Multimedia Appendix 3：心理・行動指標の介入後値および変化量（元表5）、SMSテキストメッセージングの受容性（元表10）, SMS配信頻度・タイミング・期間（元表11）。

表示を減らす

思考時間: 2 〜 3 秒

はい。この構成は**おおむね適切**だと思います。本文の図表数を抑えつつ、介入内容・探索的アウトカム・受容性評価を補足資料に整理する方針として自然です。

ただし、**Appendix 3だけは内容の性質がやや混在している**ため、少し整理したほうが読み手には親切です。

全体評価

Multimedia Appendix 1

介入した生活習慣領域と使用数／メッセージの基盤としたBCTsと使用数

これは適切です。
介入設計の「構成要素」を示す資料なので、本文に詳細を置かず、補足資料に回す判断は妥当です。

特に、生活習慣領域とBCTsは、本文中では「介入は複数の生活習慣領域およびBCTsに基づいて構成した」と説明し、詳細な内訳をAppendixに置く形でよいと思います。

位置づけとしては、**介入内容の設計根拠・構造を補足する資料**です。

Multimedia Appendix 2

介入群に配信したSMS一覧（12週間、週3回）

これも適切です。
SMS介入研究では、実際にどのようなメッセージが配信されたかは再現性に関わるため、Appendixに一覧として示す意義があります。

Appendix 1が「介入の設計構造」、Appendix 2が「実際の配信内容」という関係になるので、流れも明確です。

位置づけとしては、**介入内容の具体例・再現性を担保する資料**です。

Multimedia Appendix 3

心理・行動指標の介入後値および変化量／SMS受容性／SMS配信頻度・タイミング・期間

ここは、補足資料に回す内容としては妥当です。
ただし、3種類の情報が入っています。

1. 心理・行動指標の介入後値および変化量
2. SMSテキストメッセージングの受容性
3. SMS配信頻度・タイミング・期間に関する評価

いずれも本文の主要アウトカムではなく、探索的・補足的・プロセス評価的な位置づけなので、Appendixに回す判断はよいです。

ただし、**心理・行動指標**と**SMS受容性・配信条件の評価**は、少し性質が異なります。

心理・行動指標は、介入効果に関連する**副次的・探索的アウトカム**です。
一方、受容性、配信頻度、タイミング、期間は、介入の**実施可能性・受け入れやすさ・プロセス評価**に近い内容です。

そのため、Appendix 3の中で見出しを分けるとよいです。

Appendix 3の整理案

現在の構成のままでいくなら、Appendix 3の内部を次のように分けると整います。

Multimedia Appendix 3. Supplementary outcomes and process evaluation

- Table S1. Psychological and behavioral outcomes after the intervention and changes from baseline
- Table S2. Acceptability of SMS text messaging
- Table S3. Perceptions of SMS delivery frequency, timing, and intervention duration

このようにすると、Appendixを1つにまとめつつ、内容の違いが明確になります。

私なら少し気になる点

気になるのは、**Appendix 3のタイトルをどうするか**です。

「心理・行動指標」と「受容性・配信条件評価」を一つのAppendixに入れる場合、タイトルを狭くしすぎると、後半のSMS評価が浮きます。

たとえば、

Multimedia Appendix 3. Supplementary psychological and behavioral outcomes and SMS process evaluation

のようにすると、3つの表を含めても違和感が少ないです。

日本語で考えるなら、

Multimedia Appendix 3：心理・行動指標およびSMSテキストメッセージングに関するプロセス評価

くらいがよいと思います。

結論

この3分割は、かなり整理されています。

特に、

- Appendix 1：介入設計の構造
- Appendix 2：実際に配信したSMS内容
- Appendix 3：本文に収めきれない探索的アウトカム・受容性・プロセス評価

という役割分担になっており、本文表数を抑える目的にも合っています。

修正するとすれば、**Appendix 3の中で「心理・行動指標」と「SMS受容性・配信条件評価」を小見出しまたは表番号で明確に分ける**ことです。そこを整えれば、構成としては十分妥当です。
