# Supplementary material for: A Behavior Change Technique–Based Text Messaging Intervention for Obesity Among Employed Adult Men Younger Than 40 Years: Pilot Randomized Controlled Trial
Source: JMIR Form Res. 2026 Oct 20;10:e104711. doi: 10.2196/104711 (PMC13717270; doi:10.2196/104711)
Supplement: Supplementary file 3 [file formative-v10-e104711-s001.docx]

Table S1. Lifestyle domains^a^ targeted by the intervention and frequency of use

| Item | Frequency of use ^b^ |
| --- | --- |
| Diet | 13 |
| Alcohol consumption | 5 |
| Physical activity | 6 |
| Sleep | 4 |
| Psychosocial and socioeconomic factors | 9 |
| Self-monitoring | 5 |

^a^ Lifestyle domains were based on references [1,2].

^b^A single messge may include multiple lifestyle habits.

Table S2. BCTs^a^ used in the text messages and their frequency of use

| Label | Frequency of use ^b^ |
| --- | --- |
| 1.1 Goal setting (behavior) | 5 |
| 1.2 Problem solving | 5 |
| 1.3 Goal setting (outcome) | 4 |
| 1.4 Action planning | 6 |
| 2.2 Feedback on behavior | 3 |
| 2.3 Self-monitoring of behavior | 3 |
| 2.4 Self-monitoring of outcome(s) of behavior | 5 |
| 3.1 Social support (unspecified) | 2 |
| 4.1 Instruction on how to perform the behavior | 9 |
| 5.1 Information about health consequences | 6 |

^a^ BCTs: Behavior change techniques , based on references [3,4].

^b^ A single message may contain multiple behavior change techniques.

## References

1. The Japan Society for the Study of Obesity. Clinical Practice Guidelines for Obesity. Setagaya-ku, Tokyo: Life Science Publishing; 2022:161-166. ISBN:9784897754581.
2. Ministry of Health, Labour and Welfare. Standard Health Checkup and Health Guidance Program (FY 2024 Edition).
3. Michie S, Richardson M, Johnston M, et al. The behavior change technique taxonomy (v1) of 93 hierarchically clustered techniques: building an international consensus for the reporting of behavior change interventions. Ann Behav Med. 2013;46(1):81-95. PMID:23900692 doi:10.1007/s12160-013-9486-6
4. Michie S, Atkins L, West R. The Behaviour Change Wheel: A Guide to Designing Interventions. London, United Kingdom: Silverback Publishing; 2014:259-283. ISBN:9781912141005.
