# Supplementary material for: A Behavior Change Technique–Based Text Messaging Intervention for Obesity Among Employed Adult Men Younger Than 40 Years: Pilot Randomized Controlled Trial
Source: JMIR Form Res. 2026 Oct 20;10:e104711. doi: 10.2196/104711 (PMC13717270; doi:10.2196/104711)
Supplement: Supplementary file 4 [file formative-v10-e104711-s002.docx]

Table S3. List of SMS sent to the intervention group (12 weeks, three times per week)

| Delivery date | Day of the week | Message sent | Lifestyle domain ^a^ | BCTs^b^ |
| --- | --- | --- | --- | --- |
| Week 1 | Mon | Hello, [Name]. Starting today, I’ll be sending you messages and quizzes three times a week to help you lose weight at your own pace. Today, I have two things to share with you. ① Let’s set a target weight. Ideally, you should aim to lose 1% of your current weight per month. ② Starting today, let’s track your weight and step count. Weigh yourself at the same time every day. We recommend doing so in the morning and before bed. | Self-monitoring | 1.3. Goal setting (outcome)  2.3. Self-monitoring of  behavior |
|  | Wed | Hello. Do any of the following behaviors apply to you when you’re out and about? ① When parking, you look for a spot near the store entrance. You also drive around the neighborhood. ② You sit down immediately when you get on the train. ③ You tend to use elevators and escalators. You don’t really want to use the stairs.  If any of these apply to you, let’s incorporate “walking, standing, and moving” into your daily routine. | Physical activity | 1.4 Action planning |
|  | Fri | Hello. The weekend is almost here. I’m sending separate messages for those who drink alcohol and those who don’t.  [For those who drink alcohol] For snacks, we recommend vegetables, tofu, and protein-rich foods like meat and fish. Fried foods, ramen as a late-night meal, and rice balls are the enemies of weight loss. Let’s avoid them.  [For those who drink alcohol] Why not try to avoid snacking while watching TV or browsing the internet? Snacks and sweets right before bed are the enemy of weight loss. Let’s cut them out. | Diet | 1.2 Problem solving  5.1 Information about health  consequences |
| Week 2 | Mon | Hello. I have a question about beverages. When you want a change of pace or when you’ve worked up a sweat, what kind of drinks do you choose?  If you thought of fruit or vegetable juices—which are often perceived as healthy—or carbonated drinks that go down smoothly, be careful. They may contain a lot of sugar. Choose unsweetened tea or coffee instead. | Diet | 1.1 Goal setting (behavior)  2.3 Self-monitoring of  behavior |
|  | Wed | Hello. Today we’re sending you a health quiz. Please check it out at the URL below. https://pages.cm.com/6698550000/448e5307-228a-403b-a22f-67a42a2261ce  Even just walking a little while at work has health benefits. True or false?  The answer is true. Let’s move our bodies 10 minutes more than we do now. If you have a desk job, try to get up from your chair at least once an hour. For example, how about “taking the stairs to a restroom on a different floor”? We also recommend exercise that gets your heart pumping. | Physical activity | 1.2 Problem solving |
|  | Fri | Hello. Are you managing your stress? If you drink alcohol before bed to relieve fatigue, it puts a strain on your body and leads to shallow sleep . I recommend diaphragmatic breathing, light stretching, or taking a bath.  Click here for abdominal breathing instructions [[url:https://www.med.or.jp/komichi/holiday/sports_02_pop.html]] (Japan Medical Association: Key Points of Abdominal Breathing) | Alcohol consumption  Sleep | 4.1 Instruction on how to perform the behavior |
| Week 3 | Mon | Hello. Do you have anyone who supports your weight loss efforts? If someone cooks for you, ask for their help! Even if you mainly cook for yourself or eat out, try telling someone close to you about your weight loss goals.  Be sure to make use of the pamphlet we distributed (pp. 4–5), “Maintain a Regular Diet and Beware of Overeating!” You can find the pamphlet here: https://e-kennet.mhlw.go.jp/tools_bmi/ (Ministry of Health, Labour and Welfare Health Promotion Network: Obesity Prevention (Men’s Edition)) | Diet  Psychosocial and socioeconomic factors | 3.1 Social support (unspecified),  4.1 Instruction on how to  perform the behavior |
|  | Wed | Hello. Today, we’re sending you a health quiz. How much energy do you think you need each day?  You can easily calculate this using the "Daily Calorie Requirements: Estimated Energy Needs (Japan Medical Association)" tool. Please give it a try! https://www.med.or.jp/forest/health/eat/01.html | Self-monitoring | 1.1 Goal Setting (behavior)  1.3. Goal setting (outcome) |
|  | Fri | Hello. Convenience stores and supermarkets are full of temptations, aren’t they? Let’s walk right past the snacks, sugary drinks, and the fried food section by the register.  Even if you feel like going back, resist the urge! After all, this is your chance to take on the challenge of losing weight. | Diet | 1.2 Problem solving |
| Week 4 | Mon | Hello. You’re now in your fourth week of your weight loss journey. Have your diet or exercise routine changed? If you’re doing your best, give yourself a pat on the back. Even if you’re not, make sure to step on the scale every day.  And why not think about what you can add to your daily routine? Here are some specific tips: https://e-kennet.mhlw.go.jp/tools_bmi/ (Notebook for Reviewing Lifestyle Habits to Build a Healthy Body) | Psychosocial and socioeconomic factors | 2.2 Feedback on behavior |
|  | Wed | Hello, [Name]. Today, we’re sending you a health quiz. Please check it out at the URL below.  https://pages.cm.com/6698521000/88832869-4de8-44a7-8906-21e6b6fde1ca  Which of the following is your favorite lunch option when eating out? ① Katsudon set, ② Ramen and gyoza, ③Yakiniku set meal, ④ Grilled fish and simmered vegetable set meal.  If you chose a Japanese-style set meal like option ④, keep it up! Dishes like the rice bowls (options ①–③), ramen, and yakiniku are generally high in calories, fat, and salt. If these are your favorites, we recommend switching to a Japanese-style set meal. Let’s say goodbye to all-you-can-eat buffets! | Diet | 1.2 Problem solving |
|  | Friday | Hello. On your days off, after sitting and watching TV or using your smartphone, why not make time to move your body—such as by stretching your shoulders, which you don’t usually use?  After exercising, you might feel like drinking a carbonated soda or alcohol, but why not try switching to sugar-free sparkling water instead? | Diet  Physical activity | 1.1 Goal setting (behavior)  1.4 Action Planning |
| Week 5 | Mon | Hello. Are you weighing yourself? If you’ve been doing so, please keep it up. If not, let’s start again today. Weighing yourself every day helps you confirm that you haven’t gained weight, and even if you do gain a little, it’s easier to lose it. Let’s keep it up. | Psychosocial and socioeconomic factors | 2.2 Feedback on behavior |
|  | Wed | Hello. Today we’re sending you a health quiz. Please check it out via the URL.  https://pages.cm.com/6698522000/e4c2f4d0-8f30-4313-a2a7-fd8231e88472  When converted to average daily net alcohol intake, which of the following best represents the amount of alcohol consumption by men that increases the risk of lifestyle-related diseases? ①30 grams ,②40 grams, ③50 grams .  The correct answer is ②40 grams (2 standard unit). The approximate amounts of various alcoholic beverages equivalent to 20 grams of pure alcohol (1 standard unit) are as follows: beer (one 500-ml bottle), sake (180 ml), shochu (35% ABV) (180 ml), and wine (120 ml).  For more details, visit https://e-kennet.mhlw.go.jp/wp/wp-content/themes/targis_mhlw/pdf/leaf-alcohol-male.pdf?1706140800059 (Ministry of Health, Labour and Welfare: "Change Your Habits, Prepare for the Future: You Decide How to Enjoy Alcohol") | Alcohol consumption | 4.1 Instruction on how to  perform the behavior  5.1 Information about health consequences |
|  | Fri | Hello, [Name]. Five weeks have passed. Even if your weight loss slows down, let’s keep going without getting discouraged. For example, try increasing your step count by 1,000 steps or adding 5 minutes to your active time—let’s build on the efforts you’re making and the progress you’ve achieved. | Self-monitoring | 1.3 Goal setting (outcome) |
| Week 6 | Mon | Hello. Are you paying attention to nutrition labels? Nutrition Facts Table Source: "Overview of the Food Nutrition Labeling System" (Consumer Affairs Agency) (Only the "Nutrition Facts" chart from p. 3 is shown here)  If you’d like to learn more, make use of nutrition labels to help prevent metabolic syndrome! Consumer Affairs Agency https://www.caa.go.jp/policies/policy/food_labeling/nutrient_declearation/consumers/assets/food_labeling_cms206_20210519_02.pdf  is recommended. The example of potato chips (second page, left side) is easy to understand. | Diet | 1.4 Action planning |
|  | Wed | Hello. Today, we’re sending you a health quiz. Please check it out via the URL  https://pages.cm.com/6698535000/05b32a9b-da1b-403d-ad32-02ee9209dd88  Which of the following items are listed on the "Nutrition Facts" label found on commercially available foods: ①Calories, ② Carbohydrates (Sugars), or ③ Sodium, Fat, and Protein?  ①only, ① and ② only, or all three  The answer is: all of the above. If checking all of them is too much trouble, start by focusing on calories (energy). This will help prevent overeating. | Diet | 4.1 Instruction on how to perform the behavior |
|  | Fri | Hello. Weight loss doesn’t happen all at once; it progresses in stages. Even when you feel like you’re not seeing any changes, keep going. When you’re tired, take a short break and then try again. | Psychosocial and socioeconomic factors | 2.4 Self-monitoring of outcome(s) of behavior |
| Week 7 | Mon | Hello. Are you tracking your weight? If you are, please keep it up. Try using a method that works best for you, such as an app or a log sheet.  I recommend tracking your weight because it helps you understand the relationship between your daily step count, meals, and changes in your weight. | Self-monitoring | 2.2 Feedback on behavior  2.3 Self-monitoring of  behavior |
|  | Wed | Hello. Today we have a health quiz for you. Please check it out at the URL below: https://pages.cm.com/6698523000/25f7463c-339f-4f9a-9a38-7d04bffd2840  If you cut back on rice when drinking alcohol, it can serve as a meal replacement. True or false?  The correct answer is ×. Alcohol provides energy but contains almost no nutrients. For those who drink alcohol for work, I recommend declaring that you’ve “quit drinking” or limiting yourself to just the first drink when socializing. Surprisingly, many people are supportive of this. | Alcohol consumption | 1.2 Problem solving  1.4 Action planning |
|  | Fri | Hello. Please try to drink in moderation on your days off. For snacks, we recommend vegetables like edamame or soy products such as chilled tofu.  Even with the same meat or fish dishes, the calorie content varies depending on the cooking method. As an alternative to oil-based methods like deep-frying or tempura, I recommend cooking in the microwave. Don’t forget to get some exercise over the weekend! | Diet  Alcohol consumption | 1.4 Action planning |
| Week 8 | Mon | Hello. If this eating pattern sounds familiar—"skipping breakfast, soba for lunch, and yakiniku for dinner"—please make sure to eat three meals a day even while trying to lose weight! Eat one serving per meal, chew thoroughly, and aim to feel full. | Diet | 5.1 Information about health consequences |
|  | Wed | Hello. Today we’re sending you a health quiz. Please check it out via the URL  https://pages.cm.com/6698536000/38219d30-2e2e-4c64-80bd-4d799832c6bd  Which has more calories: a 500ml bottle of cola or a medium-sized glass of beer?  The correct answer is Coca-Cola. A medium-sized glass of beer (500ml) has 200 kcal, while Coca-Cola (500ml) has 225 kcal. Keep this in mind the next time you pick one up!  So, are “zero calories” and “zero carbs” safe? Even if it says “zero,” the product may still contain calories or carbohydrates. Be sure to check the nutrition facts label and watch out for excessive carbohydrate (sugar) intake. | Alcohol consumption | 5.1 Information about health consequences |
|  | Fri | Hello. It’s been two months since you started your weight loss journey. Have you noticed any changes when comparing your weight from last month to this month?  Let’s keep up the good work that led to this progress. Even if changing your diet or exercise habits feels like a hassle, why not try changing just one thing? | Self-monitoring | 2.4 Self-monitoring of outcome(s) of behavior |
| Week 9 | Mon | Hello. Are you walking instead of using cars, elevators, or escalators?  Let’s try to increase the amount of time you spend walking and your step count in your daily life. You can also track your progress using the “FUN+WALK App,” which supports the Japan Sports Agency’s “FUN+WALK PROJECT.” | Physical activity | 1.1 Goal setting (behavior)  4.1 Instruction on how to perform the behavior |
|  | Wed | Hello. Today, we’re sending you a health quiz. Please check it out at the URL below. https://pages.cm.com/6698539000/a9041214-7488-480a-a296-29e4bb29dbda  When you’re working late, which of the following dinner options is more appropriate: ① or ②?  ① Eat a full meal after getting home ,② Have a light snack before working overtime.  The correct answer is ②. It is said to be best to wait three hours after eating before going to bed. If you feel hungry after a light meal, we recommend a warm drink or soup. | Diet  Sleep | 1.1 Goal setting (behavior) |
|  | Fri | Hello. Even if you’re busy every day with work and family responsibilities, please try to make even a little time to take care of your body and mind.  Here is a YouTube video that explains how to manage stress through daily activities like bathing, eating, and sleeping—all in just 5 minutes. https://youtu.be/LoNQfE3NFpc?si=WdB4Ob_Bjc3wjClr (“Kokoro no Mimi” Ministry of Health, Labour and Welfare) | Psychosocial and socioeconomic factors | 2.4 Self-monitoring of outcome(s) of behavior |
| Week 10 | Mon | Hello. A word of caution for those with desk jobs or who sit for extended periods. Prolonged sitting is linked to future health issues such as cancer, cardiovascular disease, and diabetes.  Ideally, you should stand up and move around once every 30 minutes, but why not start by standing up once an hour? We also recommend placing a bamboo foot roller at your feet or doing stretches while seated. | Physical activity | 4.1 Instruction on how to perform the behavior  5.1 Information about health consequences |
|  | Wed | Hello, [Name]. Today, we’re sending you a health quiz. Please check it out via the URL.  https://pages.cm.com/6698540000/89c8e745-cc83-4520-8c45-e9f29b07e6d3  Is it a good idea to work out at the gym right up until bedtime? True or false?  The correct answer is ×. You should finish your gym workout at least 2 to 4 hours before bedtime and set aside some time to relax before going to sleep. | Physical activity  Sleep | 1.4 Action planning |
|  | Fri | Hello. When you feel like losing weight but find it a bit of a hassle, take a look at your midsection in the mirror.  Then, plan a reward for when you’ve slimmed down. Don’t forget to weigh yourself! | Psychosocial and socioeconomic factors | 1.3 Goal setting (outcome) |
| Week 11 | Mon | Hello. Have you seen any changes in your weight after two months of weight loss? It’s normal to hit a plateau, but let’s not get discouraged and keep going. Let’s also keep weighing yourself. | Psychosocial and socioeconomic factors | 2.4 Self-monitoring of  outcome(s) of behavior |
|  | Wed | Hello. Today, I’m sending you a quiz on eating habits. Please check it out via the URL below. https://pages.cm.com/accounts/0223e46c-e85e-4938-89e8-06d743ee636c/preview/846d28e9-dd00-43f2-910b- e8a22d49d1ba/section/0?_gl=1%2Ausc3do%2A_gcl_au%2ANjE2MDUyODk2LjE3MTI5NzU3NjU.%2A_ga%2AMTQ0OTMyMjA4OC4xNzExOTY1OTM1%2A_ga_EPL1F8JGJ8%2AMTczODU3MDU4Ni4xMDcuMS4xNzM4NTcyMjA5LjQ3LjAuMA..　Do any of the following apply to you?① Eating until I’m full , ② Eating quickly because I’m busy, ③ Liking fried foods, ④None of the above.  Regardless of which answer you chose, try to keep the following five eating habits in mind, even if you start with just one meal a day: ①Eat until you are 80% full; ② Avoid large portions of rice; ③Start your meal with vegetables; ④Chew thoroughly to feel full; ⑤ Limit fatty foods. | Diet | 4.1 Instruction on how to perform the behavior |
|  | Fri. | Hello. Have you noticed any positive changes in your body or daily life since you started incorporating mindful eating and physical activity into your routine?  For example: “I’m able to sleep at night,” “Climbing stairs isn’t as tiring anymore,” “My body has become more toned,” or “I’m spending less money unnecessarily.” Let’s take a moment to reflect. | Psychosocial and socioeconomic factors | 2.4 Self-monitoring of outcome(s) of behavior |
| Week 12 | Mon | Hello. This is the final week of the weight loss program. Have you reached a point where you don’t feel hungry even without snacking?  If you have snacks in front of you, put them away where they’re out of reach. Let’s find ways to cope with hunger. | Diet | 3.1 Social support (unspecified) |
|  | Wed | Hello. Today we’re sending you a health quiz. Please check it out at the URL below.<https://pages.cm.com/6686646000/e498a1f2-45ed-4d0b-a0c1-9fb18e0263c7>  Is there a link between getting enough sleep and weight loss? True or false?  The correct answer is True. It has been found that people who sleep less than 5 hours a day have a higher risk of developing obesity or metabolic syndrome compared to those who sleep 5 hours or more.  Make sure to get enough sleep. If you are concerned about having trouble falling asleep or experiencing light sleep, please consult a healthcare professional. Click here to read about the link between good sleep and health (8 pages total): https://e-kennet.mhlw.go.jp/tools_sleep/ (Ministry of Health, Labour and Welfare, Health Promotion Network: “A Good Wake-Up Starts with a Good Night’s Sleep: Things You Think You Know About Sleep, But Actually Don’t”) | Sleep | 4.1 Instruction on how to perform the behavior  5.1 Information about health consequences |
|  | Fri. | Hello. Thank you for your hard work over the past 12 weeks. Health is like invisible savings for the future. For those who have reached their weight loss goals, please continue these healthy habits. For those who are just one step away from their goals, please don’t worry. The fact that you took on the challenge is an achievement in itself. Let’s keep up these healthy habits. | Psychosocial and socioeconomic factors | 4.1 Instruction on how to perform the behavior |

^a^ Lifestyle domains were based on references [1,2].

^b^ BCTs: Behavior change techniques , based on references [3,4].

## References

1. The Japan Society for the Study of Obesity. *Clinical Practice Guidelines for Obesity*. Setagaya-ku, Tokyo: Life Science Publishing; 2022:161-166. [Japanese]. ISBN:9784897754581
2. Ministry of Health, Labour and Welfare. *Standard Health Checkup and Health Guidance Program (FY 2024 Edition)*. 2024:268-269. [Japanese]. <https://www.mhlw.go.jp/content/10900000/001231390.pdf> [accessed Mar 5, 2026]
3. Michie S, Richardson M, Johnston M, et al. The behavior change technique taxonomy (v1) of 93 hierarchically clustered techniques: building an international consensus for the reporting of behavior change interventions. Ann Behav Med. 2013;46(1):81-95. PMID:23900692 doi:10.1007/s12160-013-9486-6
4. Michie S, Atkins L, West R. *The Behaviour Change Wheel: A Guide to Designing Interventions*. London, United Kingdom: Silverback Publishing; 2014:259-283. ISBN:9781912141005
