# Supplementary material for: A Behavior Change Technique–Based Text Messaging Intervention for Obesity Among Employed Adult Men Younger Than 40 Years: Pilot Randomized Controlled Trial
Source: JMIR Form Res. 2026 Oct 20;10:e104711. doi: 10.2196/104711 (PMC13717270; doi:10.2196/104711)
Supplement: Supplementary file 5 [file formative-v10-e104711-s003.docx]

Table S4. Post-intervention and change scores for psychological and behavioral measures: between-group comparisons

| Outcome measures^e^ | Intervention group  median (IQR) | Control group  median (IQR) | *U* | *P* value |
| --- | --- | --- | --- | --- |
| **WEL**^a^ |  |  |  |  |
| ITT^b^ Analysis: Post-intervention Total Score | 102.0 (62.0to123.0) | 98.5 (63.0 to 102.0) | 175.0 | .48 |
| ITT Analysis: Change in Total Score (Post−Pre) | −9.0 (−40.0 to 20.0) | 0.0 (−9.0 to 11.0) | 144.0 | .78 |
| PPS^c^ Analysis: Post-intervention Total Score | 102.0 (62.0 to 123.0) | 99.5 (75.0 to 102.0) | 139.0 | .44 |
| PPS Analysis: Change in Total Score (Post−Pre) | −9.0 (−40.0 to 20.0) | −4.0 (−17.0 to 20.0) | 112.0 | .80 |
| **NGSE-J**^d^ |  |  |  |  |
| ITT Analysis: Post-intervention Total Score | 22.0 (18.0 to 27.0) | 23.5 (17.0 to 28.0) | 163.5 | .73 |
| ITT Analysis: Change in Total Score (Post−Pre) | 4.0 (−4.0 to 10.0) | 0.0 (−6.0 to 7.0) | 176.5 | .44 |
| PPS Analysis: Post-intervention Total Score | 22.0 (18.0 to 27.0) | 21.5 (17.0 to 24.0) | 138.5 | .44 |
| PPS Analysis: Change in Total Score (Post−Pre) | 4.0 (−4.0 to 10.0) | −3.5 (−8.0 to 8.0) | 136.5 | .49 |
| **Behavioral Change Stages** |  |  |  |  |
| ITT Analysis: Post-intervention Total Score | 20.0 (17.0 to 28.0) | 18.0 (14.0 to 22.0) | 190.0 | .23 |
| ITT Analysis: Change in Total Score (Post−Pre) | 3.0 (−1.0 to 12.0) | 0.0 (−4.0 to 4.0) | 189.0 | .25 |
| PPS Analysis: Post-intervention Total Score | 20.0 (17.0 to 28.0) | 18.5 (14.0 to 22.0) | 140.5 | .40 |
| PPS Analysis: Change in Total Score (Post−Pre) | 3.0 (−1.0 to 12.0) | 0.5 (−6.0 to 6.0) | 141.0 | .40 |

^a^ WEL: Weight Efficacy Lifestyle Questionnaire,

^b^ ITT: intention-to-treat,

^c^ PPS: per-protocol set

^d^ NGSE-J: Japanese version of the New General Self-Efficacy Scale

^e^Analysis sample sizes: ITT, intervention group n=17 and control group n=18; PPS, intervention group n=17 and control group n=14.

Table S5. Acceptability of the SMS text messaging intervention (intervention group, n=17)

| Item | mean(SD) | Median | Minimum | Maximum |
| --- | --- | --- | --- | --- |
| Satisfaction ^a^ | 3.5 (1.1) | 3 | 2 | 5 |
| Utilization ^b^ | 2.8 (1.2) | 3 | 1 | 5 |
| Willingness to recommend ^c^ | 3.0 (1.2) | 3 | 1 | 5 |

^a^ Satisfaction was rated from 1 (“very dissatisfied”) to 5 (“very satisfied”).

^b^ Utilization from 1 (“not used at all”) to 5 (“used to a great extent”),

^C^ Willingness to recommend the intervention from 1 (“would not recommend at all”) to 5 (“would highly recommend”).

Table S6. Appropriateness of the frequency, timing, and duration of SMS text messages (intervention group, n=17)

| Item ^a^ | Not at all appropriate, n(%) | Very appropriate, n(%) |
| --- | --- | --- |
| Frequency | 1 (5.9) | 16 (94.1) |
| Timing | 3 (17.6) | 14 (82.4) |
| Duration | 1 (5.9) | 16 (94.1) |

^a^ Each item had 2 response options (“not at all appropriate” or “very appropriate”).
